# Performance Analysis of IoT and Long-Range Radio-Based Sensor Node and Gateway Architecture for Solid Waste Management

**DOI:** 10.3390/s21082774

**Published:** 2021-04-14

**Authors:** Shaik Vaseem Akram, Rajesh Singh, Mohammed A. AlZain, Anita Gehlot, Mamoon Rashid, Osama S. Faragallah, Walid El-Shafai, Deepak Prashar

**Affiliations:** 1 School of Electronics and Electrical Engineering, Lovely Professional University, Phagwara 144411, India; vaseem.11814442@lpu.in (S.V.A.); rajesh.23402@lpu.co.in (R.S.); anita.23401@lpu.co.in (A.G.); 2 Department of Information Technology, College of Computers and Information Technology, Taif University, P.O. Box 11099, Taif 21944, Saudi Arabia; o.salah@tu.edu.sa; 3 School of Computer Science and Engineering, Lovely Professional University, Jalandhar 144411, India; deepak.prashar@lpu.co.in; 4 Department Electronics and Electrical Communications, Faculty of Electronic Engineering, Menoufia University, Menouf 32952, Egypt; walid.elshafai@el-eng.menofia.edu.eg

**Keywords:** cloud server, customized sensor node, customized gateway, FLoRa simulation, LoRa range radio, solid waste management

## Abstract

Long-range radio (LoRa) communication is a widespread communication protocol that offers long range transmission and low data rates with minimum power consumption. In the context of solid waste management, only a low amount of data needs to be sent to the remote server. With this advantage, we proposed architecture for designing and developing a customized sensor node and gateway based on LoRa technology for realizing the filling level of the bins with minimal energy consumption. We evaluated the energy consumption of the proposed architecture by simulating it on the Framework for LoRa (FLoRa) simulation by varying distinct fundamental parameters of LoRa communication. This paper also provides the distinct evaluation metrics of the the long-range data rate, time on-air (ToA), LoRa sensitivity, link budget, and battery life of sensor node. Finally, the paper concludes with a real-time experimental setup, where we can receive the sensor data on the cloud server with a customized sensor node and gateway.

## 1. Introduction

Waste management is the primary public utility for every community to ensure the protection of public health and the environment [1]. According to United Nations by 2025, 66% of the global population will be residing in urban cities, in comparison to 54% currently residing in the urban cities. It also estimates that the material consumption in cities will accelerate to nearly 90 billion tonnes by 2050, contrasted with 40 billion tonnes in 2010 [2]. The consumption of material significantly spikes up the generation of waste. The improper management, inadequate collection of waste, and dumping in the open-air adversely affect the environment [3]. However, the advancement in technology has progressively encouraged researchers to integrate the technology that can lower the effect of solid waste on the atmosphere [4]. At present, the Internet plays an imperative role in technological development and recent advancement in digital technologies had a tremendous impact on everyday lives, such as the Internet of Things (IoT) [5]. IoT is enhancing the quality of life as it authorizes the idea of smart cities by enhancing the interconnection between humans and objects [6]. The implementation of IoT in multiple sectors, such as as smart cities, intelligent wearable, smart homes, smart grids, and smart industries, have realized the real-time monitoring and controlling system [7]. The existing studies have primarily concentrated on the implementation of IoT in waste management for real-time monitoring and collection of garbage data [8]. In the waste collection system, one of the most promising technology is IoT [9,10], as IoT can track and handle the waste information from everywhere in the world over Internet Protocol (IP) [8]. In IoT, the role of wireless communication is crucial as the connectivity should be reliable and stable for continuous monitoring of the system [11]. At present, IoT is demanding minimum power consumption and long-range transmission features for wireless communication as the sensor nodes are energy-constrained devices [12]. With the evolution of Long-range (LoRa), the requirement of IoT is achieved as LoRa delivers long-range coverage with minimum power consumption [13]. The motivation of our study comes from Reference [14], in which a LoRa (Long-range) communication protocol is employed for the implementation of a smart waste management system to transmits real-time filling data with minimum energy consumption. With this advantage, LoRa Wide Area Network (LoRaWAN)-based IoT architecture is proposed with customized nodes for real-time monitoring of the bins. A customized sensor node and gateway are designed based on LoRa modulation technology for providing energy-efficient and stable connection establishment between the nodes and cloud server.

The main contribution of this study is divided as follows:

- We proposed architecture for designing and developing a customized sensor node and gateway based on LoRa technology for realizing the filling level of the bins with minimal energy consumption.
- We evaluated the energy consumption of the proposed architecture by simulating it on the Framework for LoRa (FLoRa) simulation by varying distinct fundamental parameters of LoRa communication.
- We provided the distinct evaluation metrics of the long-range data rate, time on-air (ToA), LoRa sensitivity, link budget, and battery life of sensor node.
- We concluded with a real-time experimental setup, where we received the sensor data on the cloud server with a customized sensor node and gateway.

The rest of the paper is categorized into various sections as defined. Section 2 addresses the work that has already been done in the field of solid waste management. Section 3 covers the overview of long range (LoRa) including evaluation metrics of LoRa. Section 4 covers the hardware implementation, where the design and development of customized sensor node and gateway are discussed. Section 5 covers proposed LoRa-based architecture for solid waste management. Performance analysis of LoRa is covered in Section 6. Result analysis are discussed in the Section 7, and the conclusion of this paper is finally expressed in Section 8.

## 2. Related Work

Smart waste management, in general, adopts advanced technology for enhancing the collection of waste management. The wireless technologies including wireless-fidelity (Wi-Fi), global Packet for Radio Service (GPRS), radio frequency identification (RFID), and global positioning system (GPS) are implemented for resolving the distinct issues in solid waste management. In regarding these many researchers have implemented the wireless sensor network (WSN) for monitoring the bin status wirelessly [15,16,17]. WSN implemented for scrutinizing the status of the bin and applying the closest vehicle first algorithm for optimizing the routes of municipal trucks [18]. Few researchers have integrated the web server and WSN for visualizing the filling of bins [19]. For transmitting the data to the web server, multiple communication technologies are embedded, one of these is GSM/GPRS module. AA smart collection system for solid waste is established with the integration of ultrasonic sensors, GSM protocol, and RFID technology [20]. The Vehicular Ad Hoc Networks (VANETs) framework was designed to provide a system for monitoring waste collections in real-time and almost every garbage bin has a sensor node, which detects the state of bins continuously and sends the signal to nearby vehicles when the bin is full [16]. Zigbee and Message Queuing Telemetry Transport (MQTT)-based waste management systems are also established for monitoring the bins, and MQTT protocol communicates the monitoring status from the ZigBee to the server [21]. iBags is a smart waste management system that is an integration of ZigBee and RFID for identifying the bags and communicating the status of the bins to the server [22]. The drawbacks of the ZigBee and GSM/GPRS are: GSM/GPRS communication consumes power consumption, and a prepaid amount needs to be recharged for transmitting the data [23,24]. The data transmission range of ZigBee is limited to short-range; however, the transmission range can be increased by installing Zigbee repeaters. Installation of Zigbee repeaters is having a higher possibility of connectivity failure. The evolution of LPWAN technologies has widened the opportunities for implementing IoT architecture to waste management, as this technology is capable of transmitting wireless data in the absence of remote cell technology with low power consumption [25,26]. The recent studies that have integrated into the smart waste management are: LoRa prototype is embedding for the sensor nodes for maximizing waste disposal routes in the Salamanca Area by using a low electrical energy consumption [27]. A low-powered sensor node is proposed for sensing the filling status of the bin with the assistance of LoRaWAN [28]. A recent study integrated the Raspberry Pi 3 Model B+ controller unit and LoRa communication protocol for monitoring the filling status of the waste in the bin [25]. In IoT applications, most designers have opted for microcontroller boards (Arduino board), or SOC (BeagleBone, Raspberry Pi, Lopy) for the platforms to connect sensor nodes to the internet [28]. From the above literature work, it is clear that there is a need of some architecture for designing and developing a customized sensor node and gateway that will be efficient enough for realizing the filling level of the bins with minimal energy consumption.

## 3. Overview of LoRa

Long-range (LoRa) is initiated in 2012, it can perform the communication with a high link budget with low power consumption [29]. LoRa is developed for supporting the LPWAN (Low Power Wide Area Network) that utilizes the ISM (Industrial, Scientific, Medical) bands of 433 MHz (Asia), 915 MHz (North America), and 868 MHz (Europe) [13]. LoRa modulation is formed from the CSS (Chirp Spread Spectrum) that provides robustness in case of multipath propagation interferences [30]. LoRa modulation is customized on distinct parameters, like spreading factor (SF), code rate (CR), and bandwidth (BW).

- SF is the number of chirps generated by each symbol, and its range is between 7 and 12. The higher the SF value, the better the receiver will eliminate the noise from the signal. The more time it takes to deliver a packet, the greater the amount it takes to transmit the packet.
- CF is the frequency of a carrier wave that is modulated to transmit signals; SX 1278 transceiver works on a carrier frequency of 433 MHz.
- BW depicts the frequency in the spectrum band, and it is chosen from these three bands: 500 kHz, 250 kHz, or 125 kHz. Large bandwidth represents speed transmission and the small bandwidth presents long-range transmission. The main parameter of the LoRa modulation is BW. The 2SF chirps that covers the entire frequency band is represented as LoRa symbol. Initially, it begins with a sequence of upward chirps, if the highest frequency band is achieved then frequency is wrapped and there will be a rise in the frequency again from the lowest frequency.

As LoRa is established on chirp spread spectrum modulation and the transmitted data is represented as chirp signal from a frequency range from fmin to fmax. Here, there is an opportunity of configuring the symbol by varying the SF and BW parameters. According to Reference [31], one symbol will take TS of second to transmit, which is a function of SF and BW are presented in the equation below:

(1) $$\mathrm{TS}=\frac{2^{\mathrm{SF}}}{\mathrm{BW}}.$$

CR is implemented in communication for assuring short interference during transmission. In any data transmission, LoRa modulation includes a forward error correction (FEC). This is achieved by encoding 4-bit data with 5-bit, 6-bit, 7-bit, and even 8-bit redundancies. The LoRa signal can sustain small interferences with this redundancy. CR is expressed as

(2) $$\mathrm{CR}=\frac{4}{4+n},$$

where n has values in the range between 1 and 4. CR is proportional to transmission speed and inversely proportional to the time on-air (ToA) during the transmission of data. HigherCR represents high transmission speed and low ToA.

**Figure 1:**
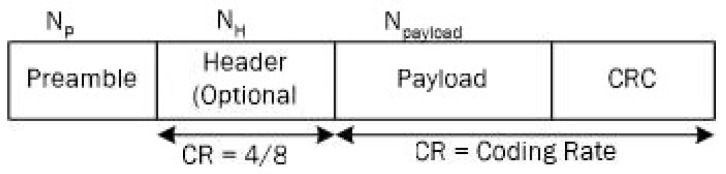
Long-range (LoRa) packet structure.

*LoRa packet structure* comprises a preamble, optional header, payload, and Cyclic Redundancy Check (CRC), as shown in Figure 1. A preamble is employed for communication between the receiver and the transmitter. The header contains the payload size and information of LoRa configuration, and it is encoded with CR = 4/8. The payload is determined with CR and CRC is transmitted at the end of the frame.

ToA is defined as the duration of time that is consumed before a receiver receives this signal. ToA of a packet is calculated with the combination of CR, SF, and BW. LoRa packet duration is defined as the combination of transmitted packet and preamble. The preamble length is expressed as follows.

(3) $$T_{preamble}=\left(n_{preamble}+4.25\right)T_{sym},$$

where npreamble indicates programmed preamble length. The payload is based upon the enabled header mode, and the number of payload symbol is calculated with this Equation (4).

(4) $$n_{payload}=8+max\left(ciel\left[\frac{\left(8\mathrm{PL}-4\mathrm{SF}+28+16\mathrm{RC}-20\mathrm{IH}\right)}{4(\mathrm{SF}-2\mathrm{DE})}\right]\left(\mathrm{CR}+4\right),0\right),$$

where PL indicates number of payload bytes, SF indicates spreading factor from 7 to 12, if IH = 0, it indicates header is enabled, if IH = 1, it indicates, if DE = 1, then Low Data Rate Optimize = 1, DE = 0; otherwise, CR is the coding rate (1 equivalent to 4/5, 4 to 4/8). The payload duration is the number of payload symbols multiplied by the symbol period, and it is expressed as:

(5) $$T_{payload}=n_{payload}*Ts.$$

The time of the air or packet is the summation of the preamble and the payload duration, and it is expressed as:

(6) $$T_{packet}=T_{preamble}+T_{payload}.$$

Bit Rate/data rate is defined as the rate at which bits are transferred from one location to another location. The bit rate (Rbit) of LoRa is expressed as:

(7) $$R_{bit}=\frac{\mathrm{SF}*\mathrm{BW}}{2^{\mathrm{SF}}*\mathrm{CR}}.$$

Sensitivity is the inherent property of a system’s ability to extract information from signals. It can also be quantified as the lowest possible signal strength that can trigger the system to its packet resolution. The first term is due to thermal noise in 1 Hz of bandwidth and can only be influenced by changing the temperature of the receiver. The second term, BW, is the receiver bandwidth. NF Is the receiver noise figure and is fixed for a given hardware implementation. Finally, SNR represents the signal to noise ratio required by the underling modulation scheme. It is the signal to noise ratio and bandwidth that are available as design variables to the LoRa designer. LoRa receiver sensitivity (S) is calculated with this expression as:

(8) $$S=-174+10\log_{10}\mathrm{BW}+\mathrm{NF}+\mathrm{SNR},$$

where S = receiver sensitivity in dB, BW = Bandwidth in KHz, NF = Noise fig. of a receiver in dB, and SNR = Signal to Noise Ratio.

*Signal-to-Noise Ratio (SNR)* is the measure of received power signal and the noise floor power level. Generally, the range of SNR is between −20 dB and +10 dB. If the range is around the +10 dB, then the received signal is less corrupted. The SNR range of LoRa is between −7.5 dB to −20 dB.

A *link budget or received power* is the summation of all the losses and gains from the transmitter, over free space to the receiver [32]. Using a simple model, the link budget can be calculated by adding transmitter power ($P_{Tx}$), receiver sensitivity (Rx), antenna gain, and free space path loss (FSPL). The link budget is expressed as:

(9) $$P_{Rx}=P_{Tx}-FSPL+G_{Tx}+G_{Rx},$$

where $P_{Rx}$ indicates received power or link budget (dBm), $P_{Tx}$ denotes transmitter power (dBm), $G_{Tx}$ denotes transmitting antenna gain (dB), $G_{Rx}$ denotes es receiver antenna gain (dB), and FSPL denotes free space path loss (dB).

Free space path loss (FSPL) is defined as the amount of energy that is lost in free space during a communication between the transmitter (Tx) and receiver end (Rx). FSPL expression derives from friis transmission equation and friis transmission equation [33] is expressed as follows:

(10) $$\frac{p_{r}}{p_{t}}={\left(\frac{\lambda }{4\pi R}\right)}^{2}G_{0t}G_{0r},$$

where $p_{r}$ and $p_{t}$ denotes power receiver and power transmitted, λ indicates wave length, and $G_{0t}G_{0r}$ directivity of the transmitting and receiving antenna. Concerning the definition of FSPL and the expression of FSPL is represented as follows:

(11) $$FSPL={\left(\frac{4\pi d}{\lambda }\right)}^{2},$$

where d denotes a distance between Tx and Rx (Meters), Tx indicates transmitter, Rx indicates receiver, f indicates the frequency in Hertz, and λ indicates wavelength.

(12) $$\lambda =\frac{c}{f};c=3*10^{8}m/s.$$

## 4. Hardware Implementation

In this section, we will discuss the importance of customization of sensor node and gateway for real-time monitoring of the filling status of the bins. Here, the sensor node and gateway are developed based on LoRa modulation; additionally, the gateway is integrated with ESP 8266 Wi-Fi module for transmitting data over the internet. In the initial stage of designing the nodes, the power consumption parameter is the key as the nodes are installed in an outdoor environment where the electrical grid network should not be utilized for powering the nodes because it increases the infrastructure cost for installing a separate electric grid network. The power consumption parameter is the key in the initial stage of designing the node, as the nodes are meant to be installed in an outdoor environment where the electrical grid network cannot be used to power the nodes because it raises the cost of infrastructure for installing separate electric grid network. Concerning powering the sensor node, we embedded the power jack for supplying power to the node through batteries. The sensor node and gateway are integrating with the +5 V and +3.3 V voltage converter to supply the appropriate voltage.

As discussed earlier in recent studies, the selection of a communication module plays a critical role in preventing the power dissipation in the nodes during transmission of the data. So, communication module is chosen with low power consumption for transmitting the data to long-range. Another major component that is taken into account for designing the node is the computing unit. The computing unit is chosen based on computational power that is a criterion for processing the acquisition data. In our case, the computing power for processing the acquired data is of low complexity because the main objective of our study is to senses the filling data. Selecting the appropriate microcontroller/microprocessor is also a vital parameter for developing an energy-efficient node. After the completion of choosing suitable components for designing the node, the next stage is subjected to integrating all these components on the single board for executing a flexible, reliable, and compatible node.

**Figure 2:**
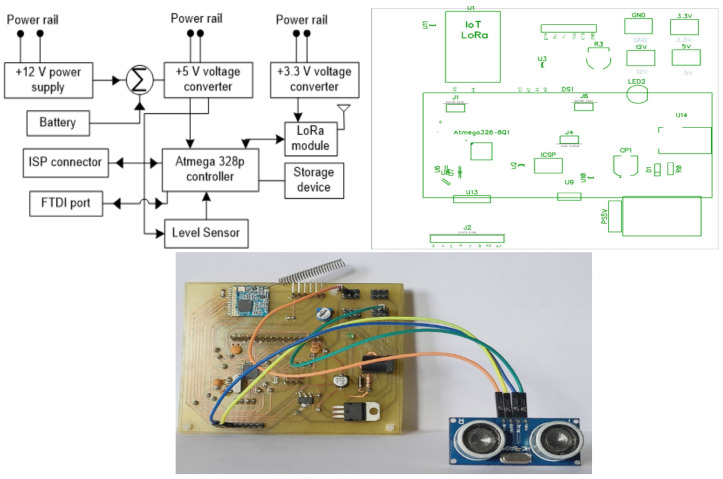
Sensor nodes.

Figure 2 illustrates the block diagram, PCB layout, and prototype of the sensor node. The node is embedding with an Atmega 328P controller and SX 1278 LoRa transceiver. In our study, the complexity of processing the data is low, we preferred the Atmega328P controller for computing the data as it consumes the least amount of current around milliamperes (mA). Here, we estimated the amount of battery required for the sensor node from Reference [34]. The sensor node sends data for every 30 min with airtime of 78.08 ms for 25-byte payload with SF7 and 125 BW. Therefore, to perform one transmission by the sensor node, it consumes 1.0962 mA of energy. If we had a battery of 2000 mAh of capacity, we could perform 1824 transmissions, i.e., performing 48 transmission per day (one transmission for every 30 min interval). With respect to battery capacity of 2000 mAh, the sensor node remains active for 38 days. If we place the battery capacity of 20,000 mAh, the sensor node performs the 18,240 transmission and the sensor node remains active for 380 days. The technical specifications of the Atmega328P microcontroller and SX1278 LoRa module are detailed in Table 1 and Table 2.

**Figure 3:**
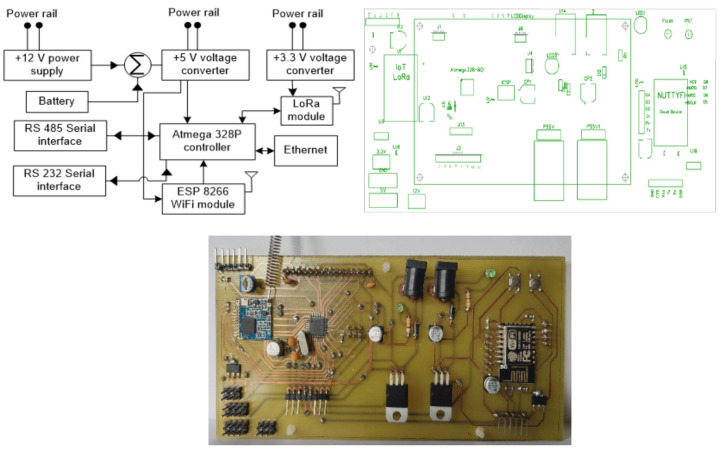
LoRa-based gateway.

Figure 3 illustrates the block diagram, schematic view, and prototype of the LoRa-based gateway. An Atmega 328P controller is built into the gateway, SX 1278 LoRa transceiver, and ESP 8266 Wi-Fi module. The objective of the gateway is for transmitting the data over multiple communication protocols, in this SX 1278 LoRa transceiver receives the data, the Atmega 328P controller triggers the ESP 8266 Wi-Fi for transmitting the data over internet protocol (IP). The technical specifications of ESP 8266 Wi-Fi are shown in Table 3.

## 5. LoRa Architecture for Waste Management

**Figure 4:**
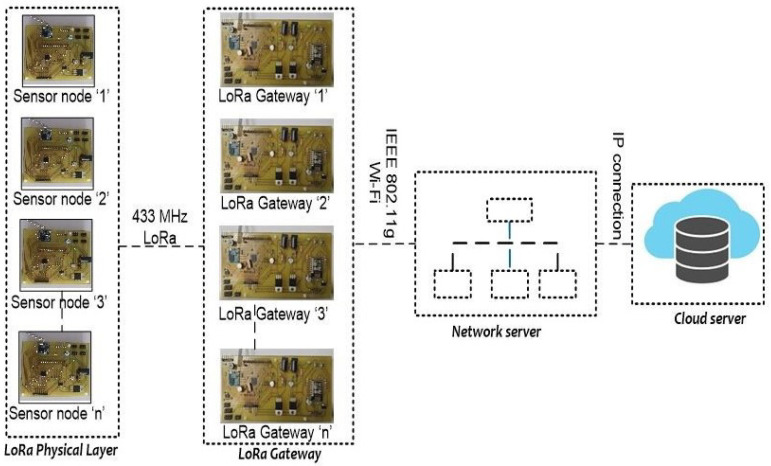
LoRa architecture for solid waste management.

After the completion of the design and development of the sensor node and gateway, we now have to implement in the real-time set up for analyzing the performance of the nodes during the transmission of the data. In order of implementing the customized node for sensing and transmitting the filling level of the bins, we are proposing architecture for waste management that is based on basic LoRa architecture and as shown in Figure 4. Physical Layer, gateway, network server, and cloud server are the key layers that exist in this architecture. The sensor nodes are part of the physical layer, the function of the sensor node is to sense the filling level of the bins and transmits the sensory data to the gateway via LoRa (Long-range).

The gateway in Figure 4 receives the messages from the sensor node to the network server. Since a transmission from a sensor node may be processed by more than single gateway, they establish a star-of-stars topology as standarised by the LoRaWAN architecture. Gateway utilizes the internet connection at their location to transmit data between the nodes and the network server. In proposed system, thhe gateways can communicate with one another via IEEE 802.11-based wireless mesh network in order of reaching the network server. This part of the system is independent of the LoRaWAN architecture. To optimize the system, we presume that the gateways have a good and direct connection to the network server.

## 6. Performance Analysis

In this section, we will be discussing the performance analysis of LoRa network. Initially, the section begins with the energy consumption of the LoRa nodes in different cases. Later on, the analysis of data rate/bit rate, LoRa sensitivity, time on air (ToA), link budget, and battery life of sensor node are elaborated.

### 6.1. Energy Consumption of Nodes

In previous studies, the simulation of LoRaWAN network is performed for studying the performance of the LoRa network by using the ns-3 module [38]. Before implementing the proposed model of waste management in real time environment, it is simulated on the virtual environment for examining the performance and capability of the model. In the virtual environment, we will analyze the LoRa network behavior in our model by varying certain parameters and aspects. FLoRa is a simulation framework, that utilizing the OMNeT++ discrete event simulation library. In spite of OMNeT++ framework, FLoRa is also based on the INET Framework, which is an open-source library for OMNeT++ and its function is to aid the procedure of experimentation for distinct network protocols [39]. In addition, the FLoRa structure ensures proper implementation of the LoRaWAN architecture [40] and a reliable LoRa radio physical layer model resulting from previous experimental findings [41]. The FLoRa architecture is convenient for simulating a complete LoRaWAN architecture that is based on star topology where it consists of four entities including LoRa nodes, gateways, a network server and application server [42].

**Figure 5:**
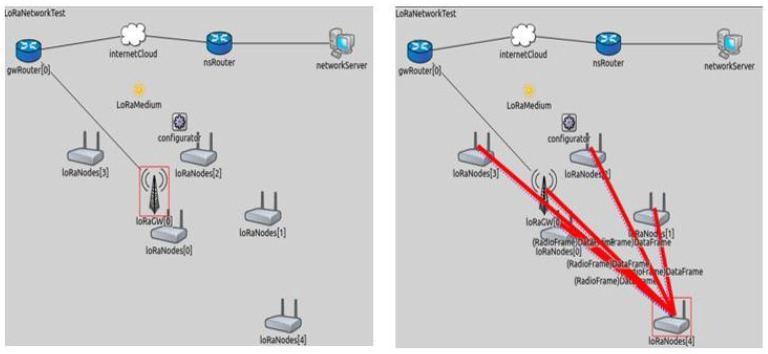
Framework for LoRa (FLoRa) graphical interface.

**Figure 6:**
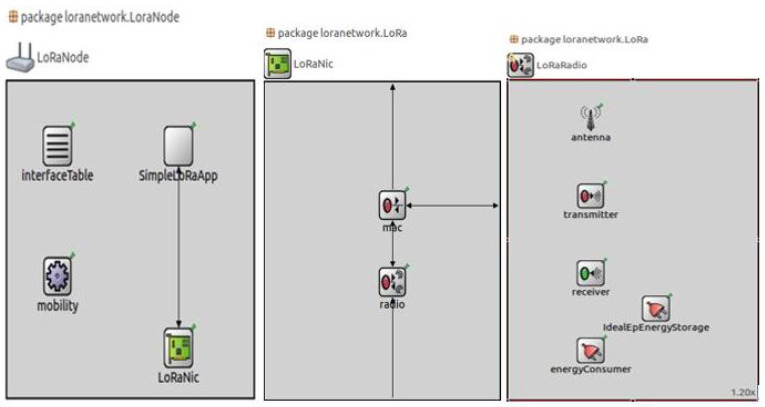
Sub-modules of LoRa Node.

Figure 5 also presents the working model of the FLoRa framework, where the LoRa nodes transmits the information to the gateway. The two nested submodules that handle the application layer (simpleLoRaApp) and the LoRa network service (LoRaNic) are shown in Figure 6. LoRaNode module includes the network module (LoRaNic) and the application module (simpleLoRaApp). LoRaNic module provides the LoRa network capability, radio module (LoRaRadio), and MAC module (LoRaMAC). In order of analyzing the behavior of LoRa network of waste management, LoRaNode is the main module in which parameters should to be modified. In this simulation we will generate the energy consumption of LoRa node by setting the parameters as CF = 433 MHz, BW = 125 KHz, SF = 7, CR = 4, No of packets = 2, network size = 480 m × 480 m, gateway distance = 320 m. number of nodes = 4, number of gateways = 1. The four nodes are represented as node ‘1’, node ‘2’, node ‘3’, and node ‘4’. The deployment type of network is select as square shape. As the network size is 480 m * 480 m, the geometry position of nodes in FLoRa environment are represented as Node 1 (263.43 m, 284.56 m), Node 2 (411.81 m, 261.54 m), Node 3 (310 m, 184.5 m), Node 4 (130 m, 184.05 m), Node 5 (444.2 m, 401.31 m), Node 6 (311.12 m, 9.70 m), Node 7 (373.51 m, 67.36 m), Node 8 (227.3 m, 383.5 m), Node 9 (374.6 m, 325.8 m), Node 10 (68.80 m, 257.9 m), Node 11 (41.8 m, 311.1 m), Node 12 (176.7 m, 399.6 m), Node 13 (373.5 m, 67.3 m), Node 14 (417.6 m, 469.7 m, Node 15 (383.5 m, 384.4 m, Node 16 (299.3 m, 374.6 m).

In case ‘1’, initially, we varied the code rate from 1 to 4, where no substantial changes are identified in the energy consumption of the LoRa node. Later on, we varied the transmission (Tx) power of the LoRa node from 2, 5, 8, 11, and 14 dBm and found the change in the energy consumption. We denoted the transmission power of the LoRa node as Tx1, Tx2, Tx3, Tx4, and Tx5. Here, remaining parameters, like CF, SF, B/W, CR, no of packets, network size, and gateway distance, are kept constant. Table 4 illustrates the energy consumption of the nodes ‘1’.

**Figure 7:**
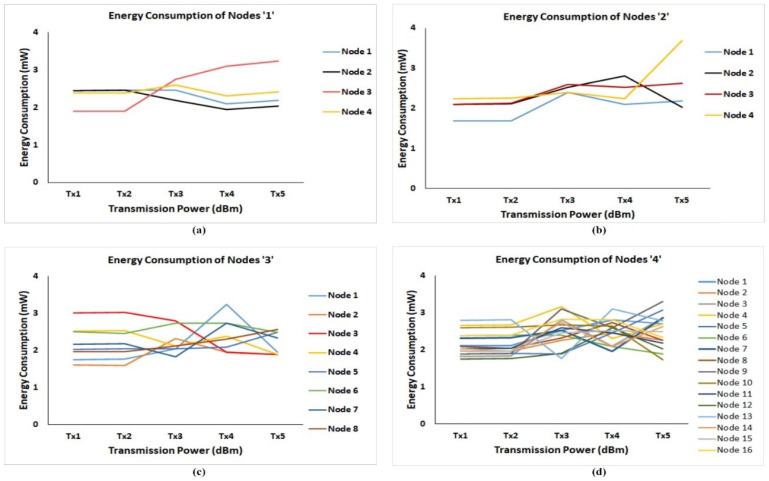
Energy consumption of nodes (**a**–**d**).

Figure 7 illustrates the graphical representation of the energy consumption of nodes of four different cases. The simulation results show that node ‘1’ and node ‘2’ has the same amount of energy consumption at the transmission (Tx) power of 2 and 5 dBm. In this case of node ‘2’, gradually, the energy consumption decreases at the Tx power of 11 dBm. Energy consumption of the node ‘3’ is suddenly increased at the Tx power of 8 dBm, i.e., from 1.90 mW to 2.74 mW. Node ‘4’ consumed the constant energy consumption for Tx power of 2 and 5 dBm; however, the energy consumption is triggered high at the Tx power of 8 dBm and suddenly the energy consumption is reduced to 2.31 mW. The part ‘a’ of Figure 7 reveals that the node ‘3’ (red color line) is consuming high energy consumption of all nodes from the Tx power of 8 dBm. The part ’a’ reveals that the node ‘3’ (red color line) is consuming high energy consumption of all nodes from the Tx power of 8 dBm. In the case ‘2’, we performed a simulation with 2 gateways by maintaining constant other parameters. The energy consumption of the 4 nodes are presented in Table 5. As the gateways have increased to two, the energy consumption of all nodes gradually decreased at the Tx power of 2 dBm.

The energy consumption of Node ‘1 decreased for the Tx of 2 dBm, 5 dBm, and 8 dBm is decreased when compared with a single gateway. In Node ‘4’, the energy consumption at all Tx power is decreased except for the Tx power of 14 dBm, where, at Tx power = 14 dBm, the energy consumption is the highest energy consumption among all nodes for all Tx power. Except for Tx power of 2 dBm and 5 dBm, the energy consumption of the Node ‘3’ is decreased at the remaining Tx power. The graphical representation of the energy consumption is represented in Figure 7b. In case ‘3’, we increased the number of nodes to 8 and the remaining parameters of the simulation are kept constant as assumed in the case ‘1’. As the number of nodes increased to the 8, the nodes are represented as node ‘1’, node ‘2’, node ‘3’, node ‘4’, node ‘5’, node ‘6’, node ‘7’, and node ‘8’. The case ‘3’ is represented as energy consumption of nodes ‘3’, and values are presented in Table 6. Figure 7c illustrates the graphical representation of case ‘3’.

The amount of energy consumption of the first four nodes has varied concerned to the previous case. In this case, Node ‘1’ has consumed a high amount of energy at the Tx power of 11 dBm, i.e., 3.25 dBm, and Node ‘2’ has consumed a low amount of energy at the Tx power of 5 dBm, i.e., 1.61 dBm. At Tx power of 14 dBm, the energy consumption for the node ‘2’, ‘3’, and ‘4’ is the same, i.e., 1.88 mW, and also for the Nodes ‘5’ and ‘6’, the energy consumption remains the same, i.e., 2.48 mW. For node ‘3’, the energy consumption gradually decreases from 3.01 mW to 1.88 mW. The energy consumption of Node ‘5’ gradually increased from 2.03 mW to 2.48 mW, and node ‘8’ also increased from 1.96 mW to 2.56 mW. The graphical representation of the energy consumption is represented in Figure 7c. In case ‘4’, we performed the simulation by increasing the number of nodes to 16, and the remaining parameters are kept constant. The case ‘4’ is represented as energy consumption of nodes ‘4’ and is presented in Table 7. The 16 nodes are represented as node ‘1’, node ‘2’, node ‘3’, node ‘4’, node ‘5’, node ‘6’, node ‘7’, node ‘8’, node ‘9’, node ‘10’, node ‘11’, node ‘12’, node ‘13’, node ‘14’, node ‘15’, and node ‘16’. The high energy consumption of nodes is recorded at Tx of 11 dBm for the Node ’13’, i.e., 3.10 mW, and low energy consumption of nodes is recorded at Tx power of 14 dBm for the Node ‘10’, i.e., 1.73 mW. Here, the energy consumption for Node ‘5’ is increased, and, for Node ‘6’, from the Tx power of 2 dBm to 14 dBm. The energy consumption for Node 1, 3, 4, 5, 6, 7, 9, 10, 11, 12, 14, and 16 are increased from Tx power of 2 dBm to 8 dBm and for node 2, 8 the energy consumption have increased from 2 dBm to 11 dBm. The graphical representation of the energy consumption is represented in Figure 7d.

### 6.2. Data Rate/Bit Rate

**Figure 8:**
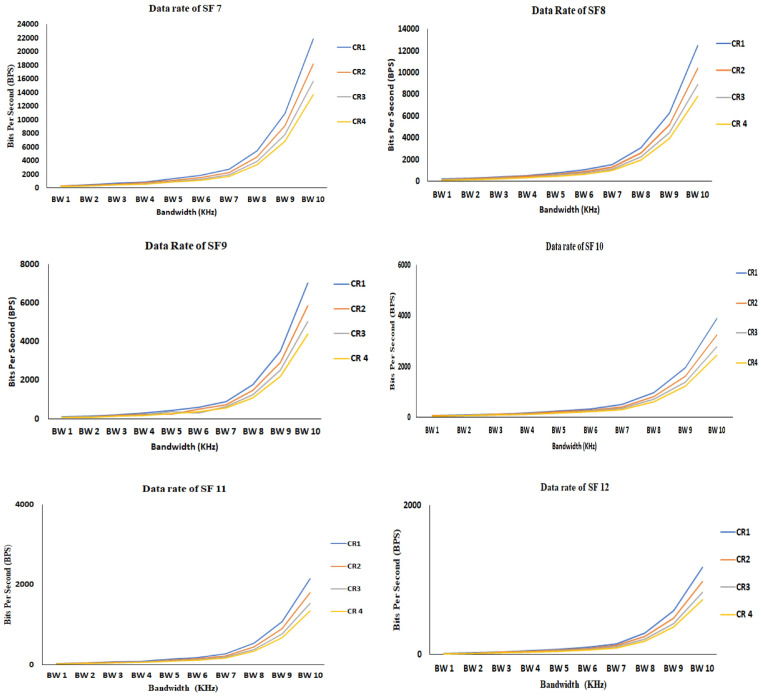
Data rate of LoRa from spreading factor (SF) 7 to SF 12.

The data rate/bit rate is defined as the quantity of bits that are transferred during transmission between transmitter and receiver. We utilized Equation (3) for calculating the data rate of the LoRa. To calculate the data rate of LoRa, the input parameters, like CR, SF, and BW, are included in the equation. Figure 8 presents the data rate of LoRa from SF 7 to SF 12. The data rate is denoted in terms of bits per second (bps). It can be observed that in every SF, the data rate is in increasing at the point of BW 7 (62.5 kHz) and it exponentially raised after the BW 8 and reached maximum at the BW 10 (500 kHz). We can observe that increasing the SF7 is inversely affecting the data rate, as the data rate declines gradually from SF 7 to SF 12. In SF 7, the data rate of the LoRa touched 22,000 bps and in SF 12 the data rate is limited to 2000 bps. An increase in SF will lead to transmitting a low amount of data during transmission, so SF 7 is the optimal SF that needs to be considered for sending a large amount of the data.

### 6.3. LoRa Sensitivity

**Figure 9:**
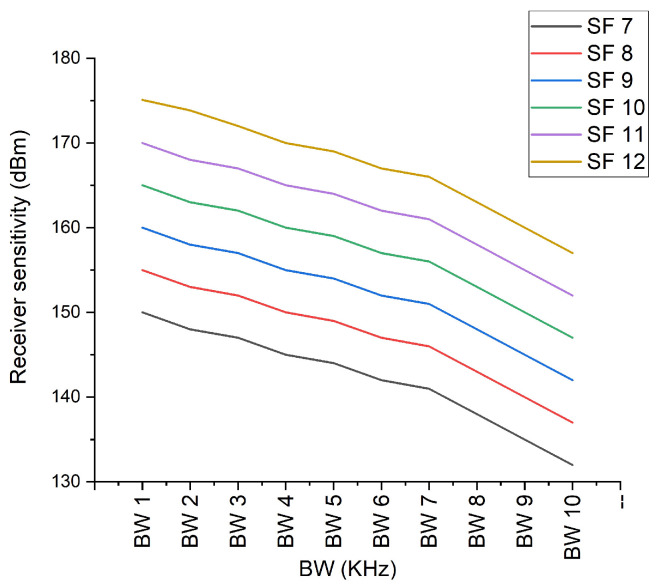
LoRa sensitivity from (SF 7–SF 12).

As discussed earlier in Section 3, the spreading factor, noise figure, and bandwidth are the input parameters for calculating the LoRa sensitivity of the receiver. Here, we utilized Equation (4) for calculating the sensitivity of the receiver. Noise fig. (SNR) value is different for distinct SF. SNR of the SF 7 is −7.5, SF 8 is −10, SF9 is −12.5, SF 10 is −15, SF 11 is 17.5, and SF 12 is −20. The above SNR value is considered for calculating the sensitivity of the receiver. As discussed in the data rate section, the same type of BW is utilized for sensitivity calculation. Generally, the sensitivity of power is in terms of a negative value; for example, −127 dBm, where the value going above this value indicates that the sensitivity is in decline. Figure 9 presents the sensitivity from the SF 7 to SF 12; the sensitivity power is highest for the SF 7 at the BW 10 (500 kHz), and the lowest sensitivity is observed for the SF 12 at the BW 1 (7.5 kHz). The sensitivity of every SF is gradually increasing after the BW 4 (20.8 kHz).

### 6.4. Time on Air (ToA)

**Figure 10:**
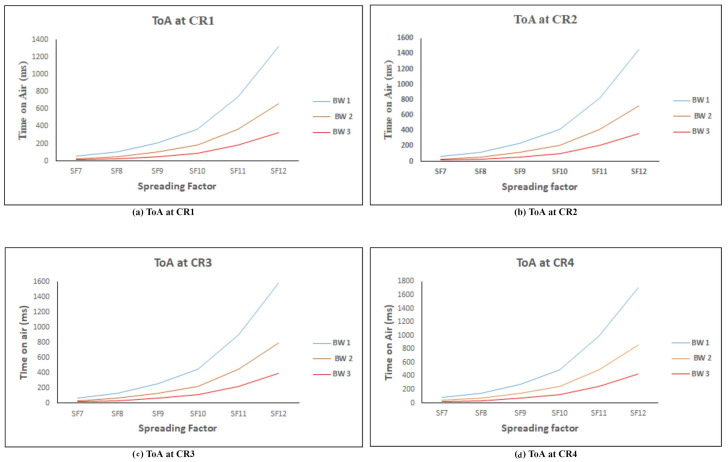
ToA for a payload of 25 bytes.

In this study, to calculate the ToA, we set certain parameters, like payload = 25 bytes, preamble = 8 symbols, CF = 433 MHz, and Tx power = 14 dBm. The tool for calculating the ToA is considered from this [43]. Here, we considered three different BW, namely 125 kHz, 250 kHz, and 500 kHz, SF from 7 to 12, and CR from 1 to 4. The calculated results are recorded in the below tabular form. ToA is calculated at the distinct CR, namely 1, 2, 3, 4, and the ToA is illustrated in Table 8, Table 9, Table 10 and Table 11. The table delivers that the ToA increases when the SF moves from 7 to 12 and ToA is reduced by half when the bandwidth increases to 125 kHz to 500 kHz. Figure 10 illustrates the ToA for the payload size of 25 bytes. The graph of the ToA at distinct CR reveals that the ToA starts increasing after the SF 9. Of all three BWs, the ToA is high for the 125 kHz BW and low for the 500 kHz BW. It is observed that the CR is directly proportional to the increase in the ToA; for example, at CR 1, the ToA of BW 1 is 56.58 ms, and, at the same BW 1, the ToA at CR 4 is 78.08 ms.

### 6.5. Link Budget

Link budget or received power is the summation of the losses and gains. The concept of the link budget is addressed in Section 3. In order to calculate the link budget, we considered Equation (11). We considered the three BW, namely 125 kHz, 250 kHz, and 500 KHz, and SF from 7 to 12.

**Figure 11:**
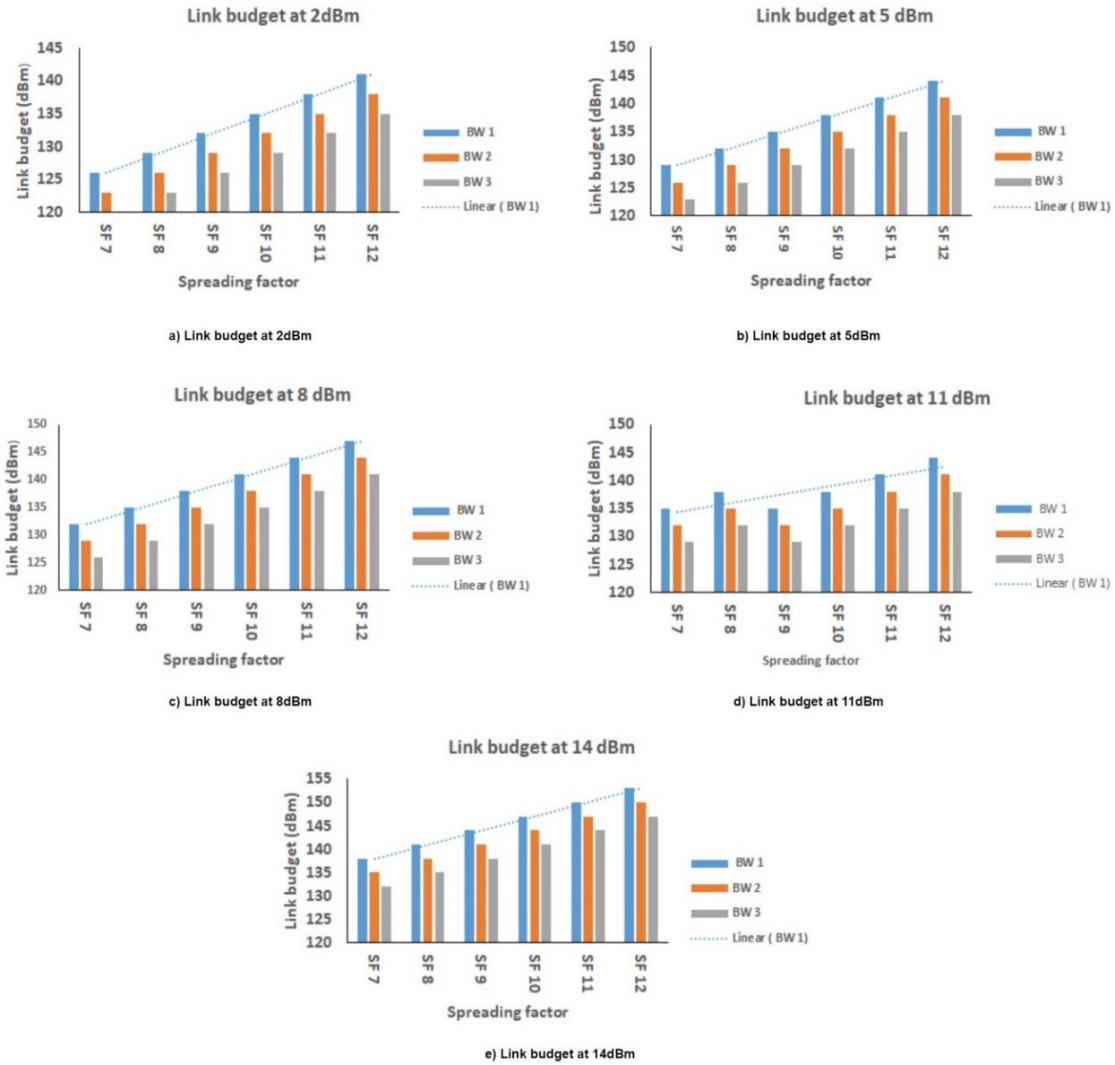
Link budget at distinct transmitter power (Tx).

As the link budget is the sum of the transmitted power (Tx) and receiver sensitivity, the increase in the Tx is leading to an increase in the link budget, and it can be observed in Figure 11. For every increment in the SF, there is also arises in the link budget. At 14 dBm, the link budget achieved maximum dBm for 125 kHz of SF 12. At 2 dBm, the link budget recorded low dBm for 500 kHz of SF7. It is concluded that the maximum link budget is achieved with high Tx power and low BW.

### 6.6. Battery Life of Sensor Node

**Figure 12:**
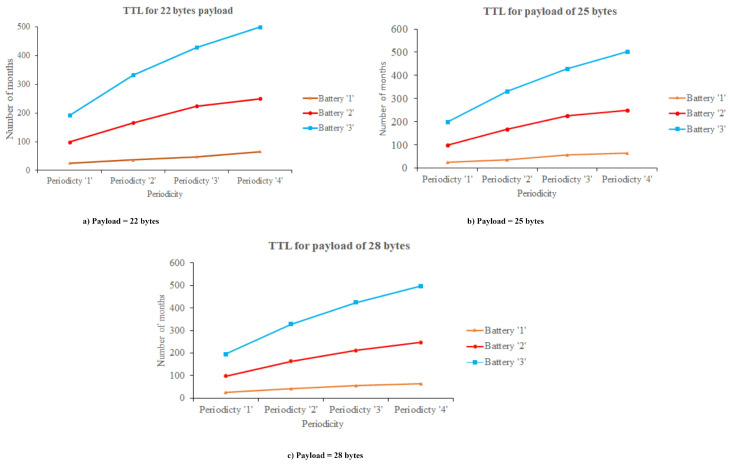
Time to live (TTL) of sensor node.

The calculating battery life of the sensor node is a necessary element in the hardware prototype. Here, we assumed the processing power of 15 mW for 5 ms during sensing value and power consumption at the sleep is 10 µW. SF 7 with 125 kHz BW and 2 dB Tx power are considered due to its optimal energy consumption during transmission. We subdivided the evaluation of battery life into three scenarios based on payload. Figure 12 presents the distinct battery life scenarios, where the battery life is represented as a time to live (TTL). In our study, we need to sense only the level measurement data, so, here, we considered a payload of 22 bytes, 25 bytes, and 28 bytes for analyzing the battery life at distinct periodicity. Here, periodicity is considered in terms of minutes likely, 15 min, 30 min, 45 min, and 60 min. The battery life of the sensor node is about months, and three distinct battery capacities are considered likely 260 mAh, 1000 mAh, and 2000 mAh Li-ion batteries. If the periodicity of sending the data from the sensor to the receiver end is 60 min, the battery life is long, and, in the case where the periodicity of sending the data of sensor is 15 min, the battery life is shorter. It is also observed that the battery life is the same for the (i) 260 mAh and 1000 mAh at a payload of 22 bytes and 25 bytes (ii) 1000 mAh at a payload of 22 bytes and 25 bytes.

## 7. Result Analysis

**Figure 13:**
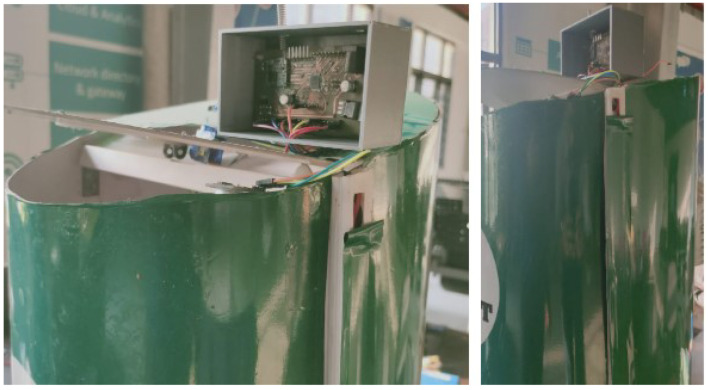
Sensor node deployed in the bins.

**Figure 14:**
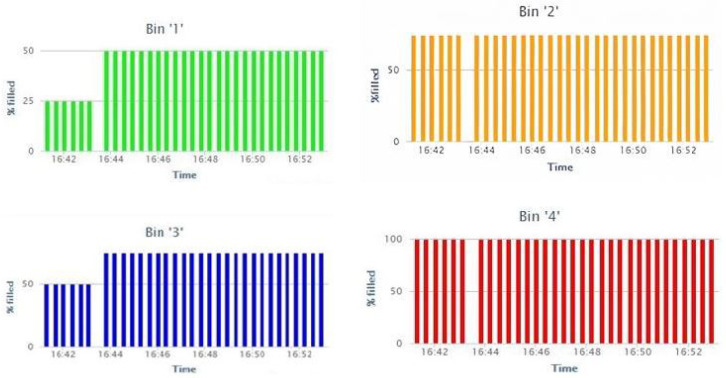
Bin status on the cloud server.

This section covers the deployment of sensor node and gateway in a real-time environment, and we will also present the sensors data that recorded on the cloud server and also a comparison of the previous studies with proposed studies is mention in detail. To evaluate the coverage of the LoRa, we deployed our sensor nodes in the bins that are located in our university. The gateway is placed at 320 m from the sensor nodes. In this study, we designed the bins for evaluating the filling levels of the bins. LoRa-based sensor node is deployed in the four bins with HC-SR 04 ultrasonic sensor (https://www.sparkfun.com/products/15569 (accessed on 7 March 2021)) with the sensor that measures the level in the bins. LoRa network is deployed in the sensor nodes according to the architecture that is proposed in Figure 4. Sensor nodes are connected with a gateway for receiving the status of the bins via the 433 MHz LoRa module. The gateway is placed at a distance of 320 m from the bins. The gateway is effectively receiving the data from the sensor node and it is logging in to the cloud server through ESP 8266 Wi-Fi module. Figure 13 illustrates the deployment of the sensor node and gateway in the real-time environment. The status of the bins that are recorded in the cloud server with graphical representation is presented in Figure 14. Distinct color representation in the graph provides the % of waste-filled in the bins. The green color represents that the bin is filled 50% and the red color represents that the bin is filled 100%. Orange and blue color represents that bins are 75% filled. With this result, we conclude that the proposed architecture based on LoRa can provide real-time data of the bins on the cloud server. Figure 14 presents that bin ‘2’ and bin ‘4’ are having constant waste for the prescribed time format.

Table 12 illustrates the comparison of previous studies focused on LoRa-based waste management with our study. The comparison is done based on the certain parameters that are illustrated in Table 12. The proposed study is having designed customized sensor node and LoRa-based gateway for realizing the LoRa-based waste management. In our implementation, we performed the simulation on FLoRa where the energy consumption of the nodes is estimated by changing distinct network parameters, like BW, CR, CF, number of nodes, and gateways. Even the distinct evaluation metrics include data rate, ToA, receiver sensitivity, link budget, and also estimated the battery life of the sensor node. The simulation of LoRa network for the waste management on the FLoRa simulator is missing in the many articles related to the waste management and also the estimated lifetime of the customized sensor node are included in our implementation. We recorded the sensor data from the sensor node with our customized sensor node and gateway in the cloud server. The data in Table 12 concludes that the customized gateway with LoRa and ESP 8266 module are implemented in limited studies. Simulation-based analysis and plot of evaluation metrics of LoRa are not addressed in any studies. Finally, the realization of the sensor data on the cloud server is observed in the few studies. Table 13 illustrates the main achievements that are achieved in the results section. The real time implementation of the proposed architecture with the customized sensor node and gateway with cloud server is the significant achievement in the results.

## 8. Conclusions

Wireless communication protocol plays key role for reliable transmission between the sensor nodes and cloud server in IoT. LoRa is one of the prominent and emergent wireless communication that meet the requirements of IoT in terms of minimum power consumption and long-range transmission. In this paper, we implemented the LoRaWAN architecture for the realizing of the real-time filling level of the bins with customized nodes. LoRa-based customized sensor nodes are deployed in the bins for establishing energy efficient nodes for transmitting the collected sensor data to the cloud server. FLoRa simulation based on LoRaWAN is performed for evaluating the energy consumption of the nodes by varying distinct parameters and also evaluated the metrics of LoRa network with plots. Finally, to show real application, an experimental set up is implemented for checking the performance of the proposed LoRa architecture for sensing the level of the bins in real-time scenario and logging the status of each of the bins in the cloud server.

## Figures and Tables

**Table 1:** SX 1278 LoRa specifications [35].

Characteristic	Specification
Frequency	433 MHz
Network topology	Point-to-Multipoint, Point-to-Point, Mesh, and Peer-to-Peer
Modulation	FSK/GFSK/MSK/LoRa
Data rate	<300 kbps
Sensitivity	−136 dBm
Output power	+20 dB
Operating voltage	1.8 V to 3.6 V
Current	Tx: 120 mA, Rx: 10.8 mA
RSSI	127 dB
Link budget	168 dB

**Table 2:** Atmega328P specifications [36].

Characteristic	Specification
Controller	8-bit microcontroller
Architecture	RISC
Programming	In-system programming
Serial interface	Master/slave SPI
PWM channels	6
Pin 6 analog pins	14 digital pins
Operating voltage	2.7 V to 5.5 V
Current	Active state: 1.5 mA at 3 V–4 MHz,
	Power-down state: 1 µA at 3 V

**Table 3:** Technical specifications of ESP 8266 [37].

Parameter	Feature
Processor	Tensilica L106 32-bit processor
IEEE standard	802.11 b/g/n
Frequency	2.4 GHz
Data rate	72 Mbps
Network Protocols	Ipv4, TCP/UDP, HTTP
Tx power	20 dBm (802.11 b), 17 dBm (802.11 g) & 14 dBm (802.11 n)
Rx sensitivity	−91 dBm (802.11 b), −75 dBm (802.11 g) & −71 dBm (802.11 n)
Operating voltage	2.5 V to 3.6 V
Current	Average: 80 mA

**Table 4:** Energy consumption of the nodes ‘1’.

Node	Tx Power = 2 dBm	Tx Power = 5 dBm	Tx Power = 8 dBm	Tx Power = 11 dBm	Tx Power = 14 dBm
1	2.45 mW	2.46 mW	2.46 mW	2.09 mW	2.18 mW
2	2.45 mW	2.46 mW	2.18 mW	1.9 mW	2.03 mW
3	1.89 mW	1.90 mW	2.74 mW	3.10 mW	3.24 mW
4	2.38 mW	2.39 mW	2.60 mW	2.31 mW	2.41 mW

**Table 5:** Energy consumption of nodes ‘2’.

Node	Tx Power = 2 dBm	Tx Power = 5 dBm	Tx Power = 8 dBm	Tx Power = 11 dBm	Tx Power = 14 dBm
1	1.68 mW	1.69 mW	2.39 mW	2.10 mW	2.78 mW
2	2.10 mW	2.11 mW	2.53 mW	2.81 mW	2.03 mW
3	2.10 mW	2.12 mW	2.60 mW	2.52 mW	2.63 mW
4	2.24 mW	2.25 mW	2.39 mW	2.24 mW	3.69 mW

**Table 6:** Energy consumption of nodes ‘3’.

Node	Tx Power = 2 dBm	Tx Power = 5 dBm	Tx Power = 8 dBm	Tx Power = 11 dBm	Tx Power = 14 dBm
1	1.75 mW	1.76 mW	2.04 mW	3.25 mW	1.95 mW
2	1.61 mW	1.59 mW	2.32 mW	1.95 mW	1.88 mW
3	3.01 mW	3.02 mW	2.88 mW	1.95 mW	1.88 mW
4	2.52 mW	2.53 mW	2.11 mW	2.38 mW	1.88 mW
5	2.03 mW	2.04 mW	2.04 mW	2.09 mW	2.48 mW
6	2.51 mW	2.46 mW	2.74 mW	2.74 mW	2.48 mW
7	2.17 mW	2.18 mW	1.83 mW	2.74 mW	2.33 mW
8	1.96 mW	1.97 mW	2.11 mW	2.31 mW	2.56 mW

**Table 7:** Energy consumption of nodes ‘4’.

Node	Tx Power = 2 dBm	Tx power =5 dBm	Tx Power = 8 dBm	Tx Power = 11 dBm	Tx Power = 14 dBm
1	2.11 mW	2.11 mW	2.53 mW	2.81 mW	2.71 mW
2	1.96 mW	1.97 mW	2.26 mW	2.46 mW	2.26 mW
3	1.82 mW	1.83 mW	2.81 mW	2.09 mW	2.86 mW
4	2.66 mW	2.67 mW	3.17 mW	2.31 mW	2.71 mW
5	1.89 mW	1.90 mW	1.89 mW	2.46 mW	3.08 mW
6	2.38 mW	2.39 mW	2.39 mW	2.09 mW	1.88 mW
7	2.31 mW	2.32 mW	2.53 mW	1.96 mW	2.86 mW
8	2.09 mW	2.04 mW	2.32 mW	2.74 mW	2.26 mW
9	1.89 mW	1.90 mW	3.10 mW	2.60 mW	3.31 mW
10	2.60 mW	2.61 mW	2.67 mW	2.64 mW	1.73 mW
11	2.03 mW	2.04 mW	2.60 mW	2.45 mW	2.18 mW
12	1.75 mW	1.76 mW	1.90 mW	2.60 mW	2.03 mW
13	2.80 mW	2.81 mW	1.76 mW	3.10 mW	2.78 mW
14	1.96 mW	1.97 mW	2.74 mW	2.09 mW	2.63 mW
15	2.03 mW	1.97 mW	2.46 mW	2.52 mW	2.48 mW
16	2.38 mW	2.39 mW	2.82 mW	2.81 mW	2.33 mW

**Table 8:** Time on-air (ToA) (ms) at code rate (CR) 1.

SF	BW 1 = 125 KHz	BW 2 = 250 KHz	BW 3 = 500 KHz
SF 7	56.58	28.29	14.14
SF 8	102.91	51.46	25.73
SF 9	205.82	102.91	51.46
SF 10	370.69	185.34	92.67
SF 11	741.38	370.69	185.34
SF 12	1318.91	659.46	329.73

**Table 9:** ToA (ms) at CR 2.

SF	BW 1 = 125 KHz	BW 2 = 250 KHz	BW 3 = 500 KHz
SF 7	63.74	31.87	15.94
SF 8	115.2	57.6	28.8
SF 9	230.4	115.2	57.6
SF 10	411.65	205.82	102.91
SF 11	823.3	411.65	205.82
SF 12	1449.98	724.99	362.5

**Table 10:** ToA (ms) at CR 3.

SF	BW 1 = 125 KHz	BW 2 = 250 KHz	BW 3 = 500 KHz
SF 7	70.91	35.46	17.73
SF 8	127.49	63.74	31.87
SF 9	254.98	127.49	63.74
SF 10	452.61	226.3	113.15
SF 11	905.22	452.61	226.3
SF 12	1581.06	790.53	395.26

**Table 11:** ToA (ms) at CR 4.

SF	BW 1 = 125 KHz	BW 2 = 250 KHz	BW 3 = 500 KHz
SF 7	78.08	39.04	19.52
SF 8	139.78	69.89	34.94
SF 9	279.55	139.78	69.89
SF 10	493.57	246.78	123.39
SF 11	987.14	493.57	246.78
SF 12	1712.13	856.06	428.03

**Table 12:** Comparison of our study with previous studies.

Research	MCU	Communication	Designed Sensor Node	Designed Gateway	Customized Node	Proof of Concept	Simulation- Based Analysis	Plot of Evaluation Metrics
[14]	Arduino Uno	SX 1272 LoRa & Waspmote	Yes	No	Yes	Yes	No	No
[27]	ATSAML21	SX 1276 LoRa	Yes	No	Yes	Yes	No	No
[28]	Atmega328P	SX 1278 LoRa	Yes	No	Yes	Yes	No	No
[29]	Atmega328P	SX 1272 LoRa	Yes	No	Yes	Yes	No	No
[44]	NA	NA	Yes	No	No	Yes	No	No
[45]	Arduino Uno	SX 1272 LoRa	No	No	No	No	No	No
[46]	Raspberry Pi3	IP67 LoRa gateway	No	Yes	Yes	Yes	No	No
[47]	Atmega328P	SX 1278 LoRa	Yes	Yes	Yes	Yes	No	No
Proposed study	Atmega328P	SX 1278 LoRa	Yes	Yes	Yes	Yes	Yes	Yes

**Table 13:** Achievements from the result section.

S.No	Parameters	Achievements
1	Energy consumption	Energy consumption of nodes are calculated in FLoRa simulation by changing distinct network parameters
2	Data rate	An increase in SF leads to transmission of a low data rate, so SF 7 is the optimal for sending a large amount of the data.
3	Sensitivity power	The sensitivity power is highest for the SF 7 at the 500 kHz.
4	ToA	ToA is good at 125 KHz and code rate 1, i.e., 56.58 ms.
5	Link budget	At 14 dBm, 125 kHz and SF 12, maximum link budget is achieved.
6	Battery life of sensor node	Periodicity of transmitting data = 60 min, the bat- tery life is long, if Periodicity of transmitting data = 15 min, the battery life is short.
7	Real time experiment set up	Realized the objective of proposed architecture in Figure 4, where the sensor data is logging into the cloud server through the customized sensor node and gateway.

## Data Availability

Not applicable.

## Abbreviations

List of abbreviations used in study:

BW	Bandwidth
CF	Carrier Frequency
CR	Code Rate
CRC	Cyclic Redundancy Check
CSS	Chirp Spread Spectrum
FEC	Forward Error Correction
FLoRa	Framework for LoRa
FSPL	Free Space Path Loss
GSM	Global System for Mobile communication
GPRS	Global packet radio service
GPS	Global Positioning System
IEEE	Institute of Electrical and Electronics Engineers
IoT	Internet of Things
IP	Internet Protocol
LoRa	Long Range
LoRaWAN	LoRa Wide Area Network
MQTT	Message Queuing Telemetry Transport
PCB	Printed Circuit Board
RFID	Radio Frequency Identification
SF	Spreading Factor
SoC	System on a Chip
ToA	Time on Air
SNIR	Signal-to-Noise Ratio
VANET	Vehicular Ad Hoc Network
Wi-Fi	Wireless Fidelity
WSN	Wireless Sensor Network
